# Head and neck symptoms during prolonged simulated weightlessness

**DOI:** 10.3389/fphys.2026.1967313

**Published:** 2026-10-30

**Authors:** Philipp H. Zimmermann, Timon Ax, Katharina Garrelfs, Edwin Mulder, Kariem Sharaf, Dipakshi D. Tare, Jens Tank, Jens P. Klussmann, Jens Jordan, Ulrich Limper

**Affiliations:** 1 Department of Otorhinolaryngology, Head and Neck Surgery, Medical Faculty, University of Cologne, Cologne, Germany; 2 School of Medicine, Western Sydney University, Sydney, NSW, Australia; 3 Department of Ophthalmology, Saarland University Medical Center, Homburg/Saar, Germany; 4 Institute of Sport Science, Leibniz University Hannover, Hannover, Germany; 5 Institute of Aerospace Medicine, German Aerospace Center, Cologne, Germany; 6 Medical Faculty, University of Zurich, Zurich, Switzerland; 7 Medical Faculty, University of Cologne, Cologne, Germany; 8 Department of Anesthesiology and Critical Care Medicine, Cologne-Merheim Medical Center, Witten/Herdecke University, Cologne, Germany

**Keywords:** cephalad fluid shift, head and neck symptoms, headache, head-down tilt bed rest, otorhinolaryngology, simulated microgravity, space medicine, vertigo

## Abstract

Microgravity exposure induces cephalad fluid shifts in astronauts, but systematically collected data on head and neck-related symptoms remain limited and largely anecdotal. We therefore performed a retrospective pooled analysis of nine mid- and long-duration six-degree head-down tilt bed rest campaigns, an established analog of microgravity-induced fluid shifts, conducted at a single research center between 2016 and 2025. Medical records of 101 healthy participants were screened for head and neck-related symptoms before, during and after bed rest. Symptoms were classified and graded using the Common Terminology Criteria for Adverse Events version 6.0, and event frequency, duration, severity and timing were analyzed with phase-specific, exposure-adjusted metrics. A total of 412 head and neck-related events were documented. During head-down tilt, 84 of 101 participants (83.2%) experienced at least one event, compared with 35 participants (34.7%) during the 15-day baseline phase preceding bed rest; overall, 91 participants (90.1%) were affected. Exposure-adjusted event rates were higher during head-down tilt (7.08 events per 100 participant-days) than during the baseline phase (3.56) or the recovery phase (2.04), and this pattern was present in all nine campaigns. Headache and vertigo were the most frequent manifestations, followed by nasal, ocular, otologic and dermatologic symptoms. Onset was analyzed separately for head-down tilt days 1–30, covered by all participants, and days 31–60, covered only by the 60-day campaigns. New events continued to occur during the second month, although at a lower rate than during the first (5.80 versus 8.21 events per 100 participant-days). With the exception of one prolonged tinnitus case, all symptoms had resolved by the end of the recovery phase. Head and neck symptoms were thus common during head-down tilt bed rest, and most events were mild and self-limiting. In the absence of a control group without bed rest, and with heterogeneous countermeasure protocols across campaigns, these descriptive findings do not establish causality. They provide a systematic reference for the design of controlled analog studies of otorhinolaryngological, ocular and dermatological responses to simulated microgravity.

## 1 Introduction

Microgravity exposure induces cephalad fluid shifts resulting from the loss of Earth’s hydrostatic pressure gradient, redistributing approximately two liters of blood and interstitial fluid towards the thoracic and cranial regions (Moore and Thornton, 1987; Jirak et al., 2022). Extensive research has examined the functional consequences of this adaptation, including spaceflight-associated neuro-ocular syndrome, altered intracranial pressure regulation, changes in cognitive performance, and impaired venous return. Many of these effects develop only after prolonged exposure and may remain subclinical for extended periods (Nelson et al., 2014; Lawley et al., 2017; Seidler et al., 2024; Limper and Jordan, 2026).

In contrast, astronauts frequently report symptoms that arise shortly after entering microgravity, predominantly affecting the head and neck region, which is particularly susceptible to the accumulation of intravascular and interstitial fluid (Hargens and Richardson, 2009). Reported symptoms range from visible facial and cervical edema (Ly et al., 2022) to nasal congestion, pressure sensations within the sinonasal cavities (Khan et al., 2025) and middle ear (Inglesby et al., 2020), pharyngeal mucosal irritation (Godard, 2025), cutaneous changes such as dryness or superficial infections (Dunn et al., 2018), and ocular discomfort characterized by dry eye, irritation, and visual strain (Ax et al., 2023; Meer et al., 2024). Importantly, functional impairment of cranial cavities and mucosal surfaces occurs in the context of immune dysregulation associated with microgravity (Marchal et al., 2024), raising concern that seemingly minor disturbances in ventilation, drainage, and epithelial barrier integrity could predispose to clinically relevant infectious complications during longer missions (Crucian et al., 2016; Cowen et al., 2024).

6° head-down tilt bed rest (HDT) is an established terrestrial analog that reproduces cephalad fluid shift and its associated physiological adaptations under controlled conditions (Hargens and Vico, 2016). During sustained HDT, cranial tissue perfusion (Roberts et al., 2021), venous return (Marshall-Goebel et al., 2019), and lymphatic drainage (Barnhart et al., 2024) are similarly altered, and participants anecdotally report head and neck-related symptoms comparable to those described during spaceflight.

These symptoms are commonly reported by crew members and by participants in microgravity analog studies, and efforts to characterize head and neck-related manifestations and their mechanisms are increasing (Kirsch et al., 1993; Diridollou et al., 2000; Barnhart et al., 2024). The available evidence from spaceflight and terrestrial analogs nevertheless rests largely on mission logs and anecdotal observations rather than on standardized, longitudinal assessment, leaving a gap in systematically collected clinical data.

We hypothesized that head and neck symptoms occur frequently during prolonged HDT and contribute to a cumulative symptom burden, despite often being regarded as minor complaints. We therefore screened the medical records of nine mid- and long-duration HDT campaigns and classified all head and neck manifestations using the Common Terminology Criteria for Adverse Events (CTCAE), describing their frequency, timing, duration and severity by study phase.

## 2 Materials and methods

### 2.1 Study design and bed rest platform

We analyzed data from nine mid- and long-duration head-down tilt bed rest campaigns conducted at the:envihab research facility of the Institute of Aerospace Medicine at the German Aerospace Center (DLR) in Cologne, Germany, between 2016 and 2025. The included studies were the second of the 2-campaign RSL-study *(Reactive Jumps in A Sledge Jump System As Countermeasure During Long-Term Bedrest)* (Kramer et al., 2017), the 2-campaign AGBRESA-study *(Artificial Gravity Bedrest Study)* (Kramer et al., 2020; Clément et al., 2022), the 4-campaign SANS-CM-study *(Spaceflight associated neuro-ocular syndrome-countermeasures)* (Moestl et al., 2025), and the first 2-campaigns of the 4-campaign SMC-study *(Sensorimotor countermeasures study)*. All campaigns followed a highly standardized experimental framework, including a 6° head-down tilt posture and comparable medical monitoring protocols including daily ward rounds by medical staff. A typical bed rest study (Traon et al., 2007) starts with a 15-day baseline phase during which participants are accommodated at the facility and remain mobile, followed by a 30 to 60-day phase of 24-hour bed rest, and concludes with a recovery phase of 14 or 15 days. During these studies, caloric intake, dietary sodium, atmosphere conditions and bedtime among other variables were highly controlled to reduce background noise.

RSL-2 compared reactive jump training in a sledge jump system with a control group without training (Kramer et al., 2017). AGBRESA allocated participants to continuous (30 min once daily) or intermittent (6 × 5 min daily) short-arm centrifugation at 1 Gz at the center of mass, or to a control group without centrifugation, with eight participants per group across the two campaigns (Kramer et al., 2020; Clément et al., 2022). SANS-CM comprised four groups: daily lower body negative pressure (25 mmHg for 6 h), moderate cycling exercise followed by 6 h of bilateral venoconstrictive thigh cuffs on six days per week, a positive control group in which head-down tilt was interrupted by 6 h of upright sitting per day, and a control group without countermeasure (Moestl et al., 2025). SMC evaluated training interventions and sensorimotor training against a control group without training. The SMC protocol has been registered at the German Clinical Trials Register (ID DRKS00033882). The countermeasures and the distribution of participants across arms are summarized in **Table 1**.

**Table 1:** Countermeasure protocols and head and neck event burden by study and intervention arm.

Study (HDT duration)/arm	Countermeasure	Targets CFS ^a^	n	Events pre/HDT/post	Rate pre/HDT days 1-30/post
RSL-2 (60 d)/training	Reactive sledge jump training	No	4	2/14/3	3.33/8.33/5.00
RSL-2 (60 d)/control	None	–	3	2/16/2	4.44/12.22/4.44
AGBRESA (60 d)/cAG	Continuous short-arm centrifugation, 30 min/day	Uncertain	8	5/43/2	4.17/9.17/1.79
AGBRESA (60 d)/iAG	Intermittent short-arm centrifugation, 6 × 5 min/day	Uncertain	8	5/32/1	4.17/9.17/0.89
AGBRESA (60 d)/control	None	–	8	6/56/4	5.00/9.17/3.57
SANS-CM (30 d)/LBNP	Lower body negative pressure 6 h/day	Yes	12	10/29/1	5.56/8.06/0.60
SANS-CM (30 d)/exercise + cuffs	Cycling exercise followed by thigh cuffs, 6 h, 6 days/week	Yes	12	8/22/4	4.44/6.11/2.38
SANS-CM (30 d)/upright sitting ^b^	Daily interruption of HDT by 6 h upright sitting	Yes	11	8/24/2	4.85/7.27/1.30
SANS-CM (30 d)/control	None	–	12	5/27/4	2.78/7.50/2.38
SMC (60 d)/training arms ^c^	Sensorimotor and training interventions	No	18	3/44/5	1.11/5.93/1.98
SMC (60 d)/control	None	–	5	0/22/1	0.00/9.33/1.43
All participants	–	–	101	54/329/29	3.56/7.76/2.04
No countermeasure (subgroup across all studies)	–	–	28	13/121/11	3.10/8.81/2.78

Event rates are given as events per 100 participant-days and were calculated from the campaign-specific duration of each phase. Because the campaigns differed in bed rest duration, the rate during head-down tilt is reported for HDT days 1–30 only, the window covered by all participants and therefore comparable across arms. Absolute event numbers refer to the complete HDT phase. CFS, cephalad fluid shift; cAG, continuous artificial gravity; HDT, head-down tilt; iAG, intermittent artificial gravity; LBNP, lower body negative pressure. ^a^ “Targets CFS” refers to the intended physiological target of the intervention, not to a demonstrated effect on head and neck symptoms. The centrifugation arms are classified as uncertain because 30 min of daily artificial gravity did not mitigate fluid-shift-related intracranial changes in the same cohort (Kramer et al., 2024). ^b^ In this arm, head-down tilt was interrupted for 6 h daily, so exposure was not continuous. ^c^ The three SMC training arms are reported together; none of the interventions targets fluid distribution.

### 2.2 Participants and cohort assembly

Participant numbers varied between campaigns due to study-specific dropouts and replacements. In the RSL-2 campaign, the medical records of four participants could not be retrieved from the archive. Complete documentation was therefore available for seven of the eleven participants who completed this campaign. AGBRESA-1 and AGBRESA-2 each contributed 12 participants. SANS-CM-1, SANS-CM-3, SANS-CM-4, and SMC-2 included 12 participants each, whereas SANS-CM-2 and SMC-1 included 11 participants due to late dropouts that were not replaced once the campaigns were underway. When early dropouts occurred and replacements were enrolled, only participants who completed the full study protocol were included in our analysis, while early dropouts were excluded. Exclusion reasons included non-adherence to the study protocol or medical conditions that could pose a disadvantage to the participant or bias study outcomes. Overall, the pooled dataset comprised 101 participants, which formed the final study cohort.

### 2.3 Medical monitoring and data collection

Medical supervision followed a standardized protocol across all campaigns and included at least one daily bedside medical ward round from each participant’s admission to the facility until discharge at the end of the recovery phase (Section 2.5). These visits included routine clinical examinations that could prompt diagnostic or therapeutic decisions at the discretion of the attending physician. At each physician–participant encounter, clinical history, physical findings, and any interventions were documented. Documentation was paper-based in the RSL-2, AGBRESA-1, AGBRESA-2, SANS-CM-1, and SANS-CM-2 campaigns, and digital in SANS-CM-3, SANS-CM-4, SMC-1, and SMC-2 campaigns.

### 2.4 Symptom selection and classification

All medical records were screened according to predefined, rule-based criteria for symptoms localized to the head and neck region during the pre-HDT, HDT, and post-HDT phases. Eligible symptoms were *a priori* defined as involving the skull, midface, ocular surface, upper aerodigestive tract, or cervical structures. Symptoms arising from deeper ocular segments or associated with spaceflight-associated neuro-ocular findings were excluded, as were gastrointestinal complaints without cranial association (e.g., reflux), lower respiratory or bronchial symptoms, and central nervous system manifestations lacking a clear head–neck focus. Headache and vertigo were retained despite their potential central origin, as both represent clinically relevant head-localized symptoms in the context of microgravity and microgravity analogs and are routinely assessed in bed rest and spaceflight studies. Pooled analyses included all participants irrespective of intervention arm. In addition, event counts and rates were summarized descriptively by study and intervention arm (**Table 1**).

### 2.5 Data extraction and database construction

A digital database was constructed to compile all medical record entries meeting the predefined head and neck symptom criteria. Symptoms were retrospectively categorized using the Common Terminology Criteria for Adverse Events (CTCAE), version 6.0 (July 22, 2025), applied as a standardized adverse-event framework to ensure consistent classification across campaigns. For each event, the CTCAE grade at first documentation (‘onset grade’) and the maximum grade reached during its course were recorded. Onset and end dates were extracted to determine event duration and temporal relation to the pre-HDT, HDT, and post-HDT phases. Events were additionally coded as resolved or ongoing at the end of the observation period. CTCAE coding and anatomical attribution were performed by a clinician with specialist training in otorhinolaryngology using predefined mapping rules between clinical descriptions and CTCAE terms. Entries that could not be unequivocally assigned were reviewed in a structured case-by-case manner to ensure consistent anatomical and clinical categorization across the dataset.

Observation windows were defined per campaign and refer to the period covered by medical documentation rather than to the data collection phases defined in the protocol publications of the individual studies. The pre-HDT phase comprised the 15 days preceding the start of head-down tilt, during which participants were accommodated at the facility and daily medical documentation was available from the day of admission onwards. For some campaigns this window is one day longer than the baseline data collection phase specified in the respective protocol publication. The HDT phase comprised HDT1 to HDT30 in the SANS-CM campaigns and HDT1 to HDT60 in RSL-2, AGBRESA and SMC. The post-HDT phase started on the first day after the end of head-down tilt, on which participants resumed the upright position, and comprised 15 days in RSL-2 and 14 days in all other campaigns. Each event was assigned to the phase in which it began. Onset during HDT was expressed as the HDT day of first documentation, with HDT1 defined as day 1. Participant-days were calculated per campaign as the number of participants multiplied by the duration of the respective phase, yielding 1,515 participant-days before, 4,650 during and 1,421 after head-down tilt.

### 2.6 Statistical analysis

Statistical analyses were performed using Microsoft Excel (Version 16.102.2; Microsoft Corporation, Redmond, WA, USA) and R (version 4.5.1; R Foundation for Statistical Computing, Vienna, Austria).Phase-specific event counts and exposure-adjusted event rates were calculated per campaign for the pre-HDT, HDT, and post-HDT phases, with rates expressed as events per 100 participant-days and participant-days derived from the campaign-specific duration of each phase (Section 2.5). Because the three phases differ in length, exposure-adjusted rates were used for all inferential comparisons, whereas event counts are reported descriptively. Differences between phases were assessed at the campaign level (n = 9) using the Friedman test as an omnibus test, followed by two-sided exact Wilcoxon signed-rank tests for the comparisons pre-HDT versus HDT and HDT versus post-HDT, with Holm correction for multiple comparisons. In the five 60-day campaigns, the number of events per participant during HDT days 1–30 and days 31–60 was additionally compared using a two-sided exact Wilcoxon signed-rank test (n = 54). This comparison addresses the time course within the bed rest phase rather than the differences between study phases and is reported without correction for multiple comparisons. Analyses by study and intervention arm were descriptive because of the small number of participants per arm. p-values are reported to three decimal places, or as p < 0.001.

## 3 Results

### 3.1 Cohort characteristics

Across nine HDT campaigns, a total of 101 healthy, non-smoking participants who had passed an intensive medical check-up prior to study inclusion completed the respective study protocols and were included in the analysis. Exclusion criteria included thrombophilia, any chronic cardiovascular, respiratory, musculoskeletal, endocrine, visceral, renal or psychiatric diseases, overweight or low exercise capacity. Participants were randomly assigned to an intervention or a control group. The cohort comprised 62 men (61.4%) and 39 women (38.6%), with a median age of 31 years (IQR 27–39; range 22–55). Mean height and weight were 174.5 ± 9.2 cm and 72.0 ± 11.7 kg, respectively, corresponding to a median BMI of 23.7 (range 18.8–28.8) (**Table 2**). The participants had no history of chronic diseases and did not take any regular prescribed medication, although contraceptives were permitted for female participants for the SANS-CM and SMC studies. The participants underwent a medical inclusion visit on the day of study start and were at that time point free of any head and neck conditions requiring treatment. During head-down tilt, 84 of the 101 participants (83.2%) experienced at least one head and neck event, compared with 35 participants (34.7%) during the preceding 15-day baseline phase. Over the entire stay, 91 participants (90.1%) were affected and 10 (9.9%) remained symptom-free (**Table 2**). Notably, no grade 4 or 5 events occurred.

**Table 2:** Baseline demographic and anthropometric characteristics of the pooled study cohort comprising 101 healthy participants across nine mid- and long-duration head-down tilt bed rest campaigns.

Characteristic	Value
Number of campaigns, n	9
Participants included in analysis, n	101
Sex, n (%)	Male 62 (61.4%); Female 39 (38.6%)
Age, years, median (IQR, range)	31 (27–39, 22–55)
Height, cm, mean ± SD	174.5 ± 9.2
Weight, kg, mean ± SD	72.0 ± 11.7
BMI, kg/m2, median (range)	23.7 (18.8–28.8)
Participants with ≥1 head and neck symptom, n (%)	91 (90.1%)
Participants without head and neck symptoms, n (%)	10 (9.9%)

Values are reported as n (%), mean ± SD, or median (interquartile range, range), as appropriate. Head and neck symptoms refer to at least one CTCAE-classified event documented during the observation period.

### 3.2 Phase-dependent event burden

Across all campaigns, 412 head and neck-related events were documented and assigned to the phase in which they began. During the pre-HDT baseline period, 54 events occurred over 1,515 participant-days, corresponding to 3.56 events per 100 participant-days. During HDT, 329 events occurred over 4,650 participant-days (7.08 events per 100 participant-days). In the post-HDT recovery phase, 29 events occurred over 1,421 participant-days (2.04 events per 100 participant-days). Exposure-adjusted event rates at the campaign level differed across the three phases (Friedman test: χ² = 13.56, p = 0.001). Rates were higher during HDT than during pre-HDT in all nine campaigns (two-sided Wilcoxon signed-rank test: V = 0, Holm-adjusted p = 0.008) and lower during post-HDT than during HDT in all nine campaigns (V = 0, Holm-adjusted p = 0.008). Campaign-level rates for all three phases are shown in **Figure 1**.

**Figure 1:**
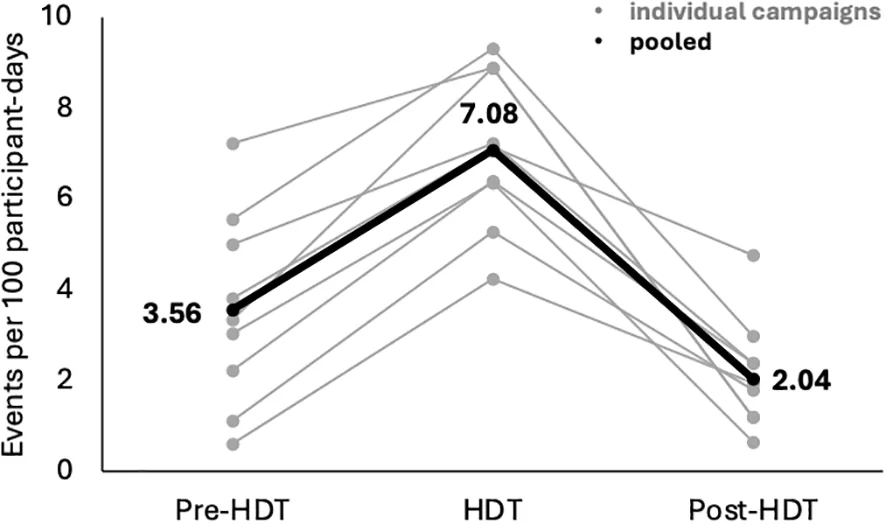
Exposure-adjusted event rates for head and neck symptoms, expressed as events per 100 participant-days, during the pre-HDT baseline phase, the HDT phase and the post-HDT recovery phase. Thin grey lines show the nine individual bed rest campaigns; the bold black line shows the pooled rate across all campaigns (54 events over 1,515 participant-days, 329 over 4,650, and 29 over 1,421, respectively). Events were assigned to the phase in which they began; participant-days were calculated from the campaign-specific duration of each phase (Section 2.5). Statistical analyses are reported in Section 3.2.

### 3.3 Event burden by study and intervention arm

Event counts and exposure-adjusted event rates by study and intervention arm are shown in **Table 1**. Because the campaigns differed in bed rest duration, rates during head-down tilt are reported for HDT days 1–30, the window covered by all participants. During this window, event rates ranged from 5.93 to 12.22 events per 100 participant-days across the eleven arms and exceeded the corresponding pre-HDT rate in every arm. The 28 participants who received no countermeasures, distributed across all four study series, showed a rate of 8.81 events per 100 participant-days during HDT days 1–30, compared with 7.76 in the full cohort. Pre-HDT event rates were low in the SMC campaigns (1.11 and 0.00 events per 100 participant-days in the training and control arms). Given group sizes of 3 to 18 participants and randomization within rather than across studies, these comparisons are descriptive and were not tested statistically.

### 3.4 Distribution of symptoms by anatomical region and phase

Event counts peaked during the HDT phase across all anatomical regions, and most events were graded as mild (**Table 3**). Headache and vertigo were the most frequent categories, with vertigo showing more frequent grade progression than headache (19.4% versus 7.7%). Nasal congestion dominated the nasal and paranasal symptoms and was documented in all phases, most frequently during HDT, whereas rhinorrhea and epistaxis were uncommon. Sinus pain occurred predominantly during HDT, lasted longer than most other categories (median 5.5 days) and showed the highest rate of grade progression (31.8%). Pharyngolaryngeal pain was mainly mild and occurred both before and during HDT, whereas oral mucositis and cheilitis were infrequent and short-lasting. Ocular symptoms were documented predominantly during HDT, with dry eye as the most frequent manifestation; eye pain occurred only during this phase. Otologic and labyrinthine symptoms were mainly documented during HDT and were predominantly mild, with one severe tinnitus episode still present at discharge. Dermatologic manifestations comprised mild facial edema during HDT, dry skin, and occasional superficial skin infections.

**Table 3:** Head and neck symptom events by CTCAE category.

CTCAE Term	Event Count			Burden		Duration	Grade		Dynamics
	Pre	HDT	Post	n	%	Median, (IQR)	onset (G1/G2/G3)	maximum (G1/G2/G3)	Progression (%)
Headache	7	81	3	91	22.1	1.0 (1.0–1.5)	(81/10/0)	(74/16/1)	7.7
Vertigo	2	56	4	62	15.0	4.0 (1.0–11.5)	(56/6/0)	(45/16/1)	19.4
Nasal congestion	10	28	3	41	10.0	2.0 (1.0–6.0)	(35/6/0)	(31/9/1)	9.8
Pharyngolaryngeal pain	10	18	3	31	7.5	2.0 (1.0–4.0)	(30/1/0)	(29/2/0)	3.2
Sinus pain	3	19	0	22	5.3	5.5 (2.0–7.0)	(19/3/0)	(12/10/0)	31.8
Neck pain	2	17	1	20	4.9	1.5 (1.0–3.0)	(16/4/0)	(15/5/0)	5.0
Dry eye	4	12	0	16	3.9	1.5 (1.0–3.2)	(16/0/0)	(16/0/0)	0.0
Ear and labyrinth disorders	0	10	2	12	2.9	2.0 (1.0–7.5)	(10/2/0)	(10/2/0)	0.0
Ear pain	0	9	1	10	2.4	3.0 (1.0–4.0)	(10/0/0)	(9/1/0)	10.0
Upper respiratory infection	2	5	3	10	2.4	4.5 (3.0–5.0)	(8/2/0)	(4/6/0)	40.0
Edema face	0	8	1	9	2.2	1.0 (1.0–2.0)	(9/0/0)	(9/0/0)	0.0
Otitis externa	0	8	1	9	2.2	11.0 (4.0–16.0)	(9/0/0)	(8/1/0)	11.1
Dry mouth	4	4	0	8	1.9	1.0 (1.0–1.8)	(8/0/0)	(8/0/0)	0.0
Dry skin	1	6	1	8	1.9	1.0 (1.0–6.2)	(8/0/0)	(8/0/0)	0.0
Eye pain	0	7	0	7	1.7	1.0 (1.0–2.5)	(7/0/0)	(6/1/0)	14.3
Tinnitus	0	5	2	7	1.7	5.0 (4.0–15.5)	(7/0/0)	(5/1/1)	28.6
Cheilitis	1	4	1	6	1.5	1.5 (1.0–2.8)	(6/0/0)	(6/0/0)	0.0
Epistaxis	0	6	0	6	1.5	1.0 (1.0–1.0)	(6/0/0)	(6/0/0)	0.0
Mucositis oral	2	4	0	6	1.5	3.0 (1.5–3.8)	(6/0/0)	(6/0/0)	0.0
Eye infection	0	5	0	5	1.2	4.0 (3.0–5.0)	(0/5/0)	(0/5/0)	0.0
Lymph node pain	0	5	0	5	1.2	1.0 (1.0–2.0)	(5/0/0)	(5/0/0)	0.0
Cough	2	0	1	3	0.7	2.0 (1.5–12.0)	(3/0/0)	(3/0/0)	0.0
Rhinorrhea	0	3	0	3	0.7	2.0 (1.5–4.0)	(3/0/0)	(3/0/0)	0.0
Anosmia	1	1	0	2	0.5	6.0 (4.0–8.0)	(2/0/0)	(2/0/0)	0.0
Middle ear inflammation	0	2	0	2	0.5	9.0 (7.0–11.0)	(1/1/0)	(1/1/0)	0.0
Skin infection	0	2	0	2	0.5	7.5 (4.2–10.8)	(2/0/0)	(2/0/0)	0.0
Dysgeusia	0	1	0	1	0.2	22.0	(1/0/0)	(1/0/0)	0.0
Dysphagia	0	0	1	1	0.2	6.0	(1/0/0)	(1/0/0)	0.0
Eyelid function disorder	1	0	0	1	0.2	1.0	(1/0/0)	(1/0/0)	0.0
Flushing	0	1	0	1	0.2	1.0	(1/0/0)	(1/0/0)	0.0
Lip infection	0	0	1	1	0.2	5.0	(1/0/0)	(1/0/0)	0.0
Pharyngitis	1	0	0	1	0.2	6.0	(0/1/0)	(0/1/0)	0.0
Sleep apnea	0	1	0	1	0.2	1.0	(1/0/0)	(1/0/0)	0.0
Trismus	0	1	0	1	0.2	1.0	(1/0/0)	(1/0/0)	0.0
Watering eyes	1	0	0	1	0.2	1.0	(1/0/0)	(1/0/0)	0.0

Phase-specific event counts refer to the phase in which the event began. Burden gives the total number of events per category and its percentage of all 412 events. Duration is given as median days with interquartile range, pooled across phases. CTCAE grades are reported as the number of events classified as grade 1, 2 or 3, at first documentation and at the maximum reached during the event; no grade 4 or 5 events occurred. Progression was defined as an increase in CTCAE grade during the course of an event. For categories with a single event, the duration is given without an interquartile range. Median duration and progression estimates for categories with n ≤ 2 should be interpreted cautiously.

### 3.5 Timing of symptom onset during HDT

Symptom onset was analyzed separately for two exposure windows because the campaigns differed in bed rest duration. HDT days 1–30 were covered by all 101 participants (3,030 participant-days), whereas HDT days 31–60 were covered only by the 54 participants of the 60-day campaigns (RSL-2, AGBRESA-1, AGBRESA-2, SMC-1 and SMC-2; 1,620 participant-days). During days 1–30, 235 events occurred, corresponding to 7.76 events per 100 participant-days; during days 31–60, 94 events occurred (5.80 per 100 participant-days). In the 30-day campaigns, 102 events occurred over 1,410 participant-days (7.23 per 100 participant-days), which is similar to the rate observed during the first 30 days of the 60-day campaigns.

Restricting the comparison to the 54 participants of the 60-day campaigns, for whom both windows comprise the same individuals and the same exposure time, 133 events began during the first month (8.21 per 100 participant-days) and 94 during the second (5.80 per 100 participant-days; Wilcoxon signed-rank test on participant-level counts, p = 0.007). This decline was not uniform across campaigns: rates were essentially unchanged in AGBRESA-1 (8.89 versus 8.89) and AGBRESA-2 (9.44 versus 9.17), whereas they fell in RSL-2 (10.00 versus 4.29), SMC-1 (6.67 versus 1.82) and SMC-2 (6.67 versus 3.89). New events were nevertheless documented throughout the exposure period in all five 60-day campaigns. Onset by symptom category and window is shown in **Table 4**. Estimates for days 31–60 are based on the 54 participants of the 60-day campaigns only.

**Table 4:** Timing of symptom onset during head-down tilt, by exposure window.

CTCAE term	Days 1–30: n	Median (IQR)	Days 31–60: n	Median (IQR)
Headache	58	14.5 (6.5–23.0)	23	44.0 (38.0–50.5)
Vertigo	46	16.0 (6.2–21.8)	10	41.0 (39.5–52.8)
Nasal congestion	22	13.5 (3.2–23.2)	6	42.5 (41.2–43.8)
Pharyngolaryngeal pain	11	13.0 (10.5–24.0)	7	43.0 (41.5–52.0)
Sinus pain	17	16.0 (9.0–23.0)	2	39.0 (37.5–40.5)
Neck pain	12	6.5 (3.8–16.5)	5	47.0 (34.0–48.0)
Dry eye	7	20.0 (17.0–23.0)	5	49.0 (39.0–51.0)
Ear and labyrinth disorders	8	4.5 (1.0–26.5)	2	56.0 (56.0–56.0)
Ear pain	4	14.0 (10.8–15.5)	5	46.0 (44.0–51.0)
Upper respiratory infection	2	23.5 (20.2–26.8)	3	59.0 (47.0–59.5)
Edema face	3	15.0 (10.0–19.5)	5	50.0 (39.0–57.0)
Otitis externa	4	22.0 (20.8–24.0)	4	45.0 (35.8–54.5)
Dry mouth	2	12.0 (7.5–16.5)	2	41.0 (38.0–44.0)
Dry skin	5	15.0 (12.0–26.0)	1	49.0
Eye pain	5	2.0 (1.0–3.0)	2	41.0 (39.0–43.0)
Tinnitus	4	18.5 (14.8–21.8)	1	57.0
Cheilitis	2	20.0 (19.5–20.5)	2	42.0 (38.0–46.0)
Epistaxis	6	11.5 (6.5–15.0)	–	–
Mucositis oral	4	18.5 (7.5–29.2)	–	–
Eye infection	2	25.0 (25.0–25.0)	3	47.0 (40.0–48.5)
Lymph node pain	3	16.0 (15.0–20.5)	2	46.5 (46.2–46.8)
Rhinorrhea	3	2.0 (1.5–5.5)	–	–
Anosmia	1	19.0	–	–
Middle ear inflammation	1	26.0	1	42.0
Skin infection	1	17.0	1	38.0
Dysgeusia	1	28.0	–	–
Flushing	1	6.0	–	–
Sleep apnea	–	–	1	38.0
Trismus	–	–	1	41.0
Total	235	7.76 events/100 pd	94	5.80 events/100 pd

Onset is expressed as the HDT day of first documentation (HDT1 = day 1). Because the campaigns differed in bed rest duration, onset is reported separately for two windows: HDT days 1–30, covered by all 101 participants (3,030 participant-days), and HDT days 31–60, covered only by the 54 participants of the 60-day campaigns (RSL-2, AGBRESA-1, AGBRESA-2, SMC-1 and SMC-2; 1,620 participant-days). Estimates for days 31–60 therefore describe a subset of the cohort. Values are median days with interquartile range; for categories with a single event in a window, only the onset day is given. Categories are listed in the order used in **Table 3**; six categories documented only before or after bed rest (cough, dysphagia, eyelid function disorder, lip infection, pharyngitis, watering eyes) are not shown. The final row gives the total number of events and the exposure-adjusted event rate per 100 participant-days for each window.

### 3.6 Medication use and supportive measures

Across all campaigns, medications and supportive measures were prescribed or recommended in response to head and neck-related symptoms. The most frequently prescribed pharmacological interventions were topical nasal decongestants and oral analgesics, with topical xylometazoline and ibuprofen representing the most commonly recorded agents. Medications targeting vestibular symptoms, including dimenhydrinate, were also frequently prescribed, whereas other pharmacological treatments were documented only sporadically. Non-pharmacological supportive measures were commonly recommended, including tea for mucosal soothing, physiotherapy, and inhalation therapy. Less frequent measures included local cooling, nasal rinses, and isolated recommendations for ear care and environmental humidification. A detailed overview of all prescribed medications and supportive measures, including absolute counts, is provided in **Table 5**.

**Table 5:** Medications and supportive measures documented in response to head and neck symptoms.

Pharmacological agent	Route	n
Xylometazoline	Topical (nasal)	61
Ibuprofen	Oral	43
Dimenhydrinate	Oral	23
Hydrocortisone	Topical (skin)	12
Panthenol	Topical (skin)	12
Amylmetacresol/Dichlorobenzyl alcohol	Topical (oral lozenge)	8
Ciprofloxacin	Topical (otic)	7
Neomycin/Polymyxin B/Hydrocortisone	Topical (otic)	6
Paracetamol (acetaminophen)	Oral	6
Chlorhexidine	Topical (oral)	3
Hydrocortisone/Clioquinol	Topical (skin)	3
Betahistine	Oral	2
Mometasone furoate	Topical (nasal)	2
Ofloxacin	Topical (eye)	2
Homeopathic preparation (vertigo symptom relief)	Oral	2
Acyclovir	Topical (skin)	1
Macrolide antibiotic	Oral	1
Naratriptan	Oral	1
Phenazone (antipyrine)	Oral	1
Procaine hydrochloride	Topical (oral)	1
Total	–	197
Supportive measure		
Tea (mucosal soothing)	–	17
Physiotherapy	–	11
Inhalation therapy	–	7
Cooling	–	2
Nasal rinses	–	2
Blow drying of ears	–	1
Humidification of atmosphere	–	1
Total		41

Listed are generic substance names, route of administration, and the number of instances (n) in which a physician explicitly documented the prescription or recommendation of the respective agent or measure, across all nine campaigns and all study phases. Multiple interventions could be documented for the same participant and for the same event. The table reflects documented clinical practice and does not imply an assessment of efficacy. In total, 197 prescriptions of pharmacological agents and 41 recommendations of supportive measures were documented.

## 4 Discussion

### 4.1 Principal findings and overall pattern of symptom occurrence

This study provides a systematic characterization of head and neck symptoms during mid- and long-duration 6° HDT bed rest, pooling data from nine campaigns conducted at DLR’s:envihab research facility. Of 101 healthy participants, 84 (83.2%) experienced at least one event during head-down tilt, compared with 35 (34.7%) during the preceding baseline phase, and both event counts and exposure-adjusted rates peaked during HDT. New events continued to occur during the second month of bed rest in all five 60-day campaigns, but at a lower rate than during the first month (5.80 versus 8.21 events per 100 participant-days), so that the symptom burden attenuated over time rather than remaining constant. The decline was not uniform: rates remained stable in the two AGBRESA campaigns and fell markedly in RSL-2 and the two SMC campaigns, and with five campaigns and small event numbers we cannot distinguish protocol-specific factors from differences between participant samples or from chance. Head and neck symptoms are thus neither confined to an initial adaptation phase nor constant over time, which argues for medical monitoring beyond the first weeks of exposure. Estimates beyond HDT day 30 derive from the 54 participants of the 60-day campaigns and rest on small event counts per symptom category.

The event rate during recovery (2.04 events per 100 participant-days) was lower than during the baseline phase (3.56). Two features of the data contribute to this asymmetry. First, events were assigned to the phase in which they began: 22 of the 329 HDT events persisted beyond the end of bed rest, so that their symptomatic days during recovery were attributed to the HDT phase, whereas 13 of the 54 baseline events extended into HDT. Because assignment by onset shifts long-lasting events towards earlier phases, and recovery is the final observation period, this systematically lowers the apparent event rate after bed rest. Second, the symptom profile differed between phases: upper airway symptoms accounted for 29 of 54 baseline events (54%), compared with 74 of 329 events during HDT (22%) and 10 of 29 during recovery (34%). This is consistent with respiratory symptoms acquired before admission becoming manifest during the baseline phase, whereas the access-restricted environment of the facility reduced exposure to new pathogens thereafter. Onset was otherwise evenly distributed across the 15 baseline days, giving no indication of increased symptom ascertainment at the admission examination.

### 4.2 Non-specific pain events of the head and neck

Non-specific pain episodes constituted the largest symptom subgroup. Headache was the most frequently reported manifestation and was typically mild, short-lived and described as holocephalic or frontally accentuated, with a median onset predominantly in the first month of HDT (**Tables 3** and **4**). Migraine-typical features were rarely documented, although systematic headache phenotyping was not performed. Headache during spaceflight and HDT bed rest has been well characterized previously, with data suggesting a shift from migraine-like presentations early after exposure towards a subsequent predominance of tension-type headache (Feuerecker et al., 2016; Oosterhout et al., 2024). The temporal pattern and clinical characteristics observed in the present cohort are compatible with a predominance of tension-type headache. However, definitive classification is limited by the absence of headache-specific diagnostic instruments. Despite their high frequency, headache episodes were generally of low intensity, short-lived and only rarely interfered with participants’ daily activities.

Neck pain was also among the more frequently reported symptoms and generally of mild intensity. With a median onset predominantly in the first month of HDT, it tended to occur earlier than the onset of headache, but was also of short duration (**Tables 3** and **4**). Musculoskeletal pain of the thoracic and lumbar spine is comparably well characterized in both spaceflight and HDT (Burkhart et al., 2019; Martino et al., 2022), though the cervical spine has received less attention (Belavý et al., 2022). On Earth, cervical musculoskeletal pain is related to migraines and also tension-type headaches (Al-Khazali et al., 2024). Neck pain was frequently documented during HDT. Whether this reflects HDT-specific cervical unloading or the general effects of prolonged bed confinement cannot be determined from the present data. The observation nevertheless suggests that cervical symptoms merit systematic assessment in future analog studies.

Sinus pain occurred predominantly during HDT bed rest and temporally overlapped with nasal congestion (**Tables 3**, **4**). This temporal pattern is compatible with a headward fluid-shift–related sinonasal mucosal edema and an impaired ventilation or drainage, as previously reported in spaceflight and ground-based analog studies (Marshburn et al., 2020; Seidler et al., 2024; Khan et al., 2025). Additional symptoms attributable to nasal mucosal irritation, such as epistaxis, rhinorrhea, and isolated anosmia, were infrequent, uniformly mild, and self-limited.

Eye pain presented as pressure-like periorbital discomfort with an earlier onset than other head and neck pain symptoms and a short-lived median duration of one day (**Tables 3**, **4**). Although ocular complaints are frequently reported during spaceflight, published data predominantly describe surface-related symptoms such as irritation, foreign-body sensation, tearing, and dry eye (Meer et al., 2024). In contrast, to our knowledge, the pressure-like periorbital pain observed in the present cohort represents a symptom phenotype that has not been systematically characterized in spaceflight or microgravity-analog studies (Harris et al., 1997). A sinonasal mechanism remains plausible; however, the earlier onset relative to nasal congestion in our data set suggests that alternative explanations should also be considered, including primary headache disorders, musculoskeletal or cervicogenic mechanisms, facial pain syndromes, and neuropathic pain (Burnham, 1949; Brazis et al., 2002).

### 4.3 Upper aerodigestive tract manifestations

Amongst the symptoms affecting the upper aerodigestive tract and oral mucosa, non-specific pharyngolaryngeal pain was most frequently reported during HDT. Other manifestations including upper respiratory infection, dry mouth, oral mucositis, dysgeusia and dysphagia were either infrequent, mild, and short-lived, or with minimal progression (**Table 3**). This pattern suggests transient functional disturbances rather than overt inflammatory or structural pathology. Narrative reviews of ENT conditions during spaceflight similarly describe throat and mucosal complaints as sporadic and generally self-limiting, likely reflecting fluid redistribution, mucosal congestion, and environmental factors rather than primary disease processes (Godard, 2025). Painful cervical lymphadenopathy was observed in a small number of cases without clear evidence of infection and is best interpreted as non-specific and not clearly attributable to HDT exposure (**Table 3**; Gaddey and Riegel, 2016).

### 4.4 Otologic and vestibular manifestations

Otologic symptoms were documented predominantly during HDT and included ear pressure with subjective hearing reduction, nonspecific ear pain, otitis media, and tinnitus (**Tables 3**, **4**). Otitis externa is discussed in the context of dermatological manifestations. While vestibular and cochlear adaptations to microgravity and HDT have been extensively characterized, effects on the external and middle ear have received less systematic attention (Hallgren et al., 2015; Reschke et al., 2017; Avcı, 2024). In the present cohort, otologic symptoms are compatible with altered Eustachian tube and middle ear mucosal function under headward fluid shift conditions, potentially affecting pressure regulation and ventilation (Inglesby et al., 2020). Tinnitus was observed mainly during HDT and recovery and was generally mild and self-limiting, although one participant experienced a prolonged and functionally relevant exacerbation. As HDT is not associated with excessive noise exposure, contributors other than noise-induced hearing loss such as psychological stress, isolation, or altered middle or inner ear function should be considered (Fabrizio-Stover et al., 2024).

Vertigo represented a prominent symptom category, occurring predominantly during the HDT phase and constituting approximately 17.0% of all events observed during HDT (**Tables 3**, **4**). Participants typically reported positional, attack-like rotational dizziness triggered by changes in head position and resolving at rest, often accompanied by persistent background dizziness. This clinical presentation is compatible with a benign paroxysmal positional vertigo (BPPV)-like phenotype, which in terrestrial settings is commonly attributed to otoconial displacement within the semicircular canals (Kim et al., 2021). However, definitive attribution of these symptoms to classical benign paroxysmal positional vertigo remains uncertain in the present study. Formal canal-specific positional testing was not performed, as standard diagnostic maneuvers are not feasible under HDT conditions. Repositioning maneuvers, including modified Epley-type techniques, were documented in individual cases as part of routine supportive care, but were not recorded systematically and are therefore not listed in **Table 5**. Together with the absence of standardized BPPV testing, this precludes any assessment of their efficacy. A mechanistic association between prolonged bed rest and BPPV-like vertigo has previously been reported in a 90-day HDT study, in which lateral canal BPPV was diagnosed between days 17 and 42 of bed rest exposure (Wang et al., 2021). In addition to potential peripheral contributors, central vestibular adaptations and sensory reweighting during prolonged unloading have been described in HDT studies (Yuan et al., 2018). Although vestibular disturbances are well documented during and after spaceflight, most reports describe non-paroxysmal vertigo, imbalance, or motion sickness-like symptoms rather than classical BPPV-like episodes (Bacal et al., 2003; Karmali and Shelhamer, 2010; Reschke et al., 2017). Taken together, the vertigo events observed in the present cohort may reflect a combination of peripheral and central vestibular processes rather than unequivocal canalolithiasis.

### 4.5 Ocular and dermatologic manifestations

Dry eye accounted for 16 of all 412 events (3.9%) and for 12 of 329 events during HDT (3.6%) (**Tables 3**, **4**). Potential contributing factors include environmental conditions such as air conditioning and screen time, altered blinking behavior, changes in tear film spread, composition and drainage, and the headward fluid shift (Sahlin and Chen, 1997; Ax et al., 2023). Dry eye symptoms are reported by ~15 to 30% of astronauts and are thus relatively commonplace (Sampige et al., 2025). Factors unique to spaceflight such as floating dust particles and lack of gravity-assisted tear drainage might account for the higher prevalence compared with HDT studies (Hurwitz et al., 1975; Chavis et al., 1978; Ax et al., 2025a). We observed several cutaneous and epithelial manifestations during HDT, including dry facial skin, localized superficial skin infection, cheilitis, dryness of the external auditory canal, otitis externa, and mild facial edema (**Tables 3**, **4**). Facial edema is a well-known clinical sign of cephalad fluid shift in microgravity and has been characterized in spaceflight and HDT settings (Diridollou et al., 2000; Norsk, 2005; Norsk et al., 2015). The other observed epithelial symptoms could be compatible with the hypothesis that HDT-related cephalad fluid redistribution contributes to cutaneous barrier stress and hence increased epithelial vulnerability. Mechanistic studies from spaceflight demonstrate alterations to cell signaling and differentiation as well as a disrupted junctional architecture, and immune modulation in human skin, with implications for barrier function (Caswell and Eshelby, 2022; Park et al., 2024). Comparable data under HDT conditions remain sparse. For otitis externa, a plausible mechanism is compromised epithelial barrier integrity combined with reduced ventilation and increased local moisture, such as prolonged or recurrent occlusion by headphones or earplugs, residual moisture after showering, or contact with wet hair, a well-established risk constellation for external auditory canal inflammation in terrestrial populations (Sander, 2001).

### 4.6 Medication use and supportive measures

The pattern of prescribed medications and supportive measures observed during HDT predominantly reflects symptom-oriented management of mild to moderate head and neck complaints rather than treatment of severe pathology. Frequently documented interventions included topical nasal decongestants, oral analgesics, and anti-vertiginous agents, complemented by non-pharmacological measures such as herbal tea for mucosal soothing (without altering the regulated fluid intake), physiotherapy, inhalation therapy, and local supportive care. A comparable profile of medication use has been described during long-duration spaceflight, identifying analgesics, agents targeting nasal congestion and allergy, and medications for vestibular symptoms among the most frequently used drug classes aboard the International Space Station (Wotring, 2015). The similarity between HDT-based prescriptions and reported in-flight medication use is consistent with, but does not demonstrate, a comparable pattern of symptom-driven medical needs. The predominance of conservative, symptomatic, and supportive approaches indicates that many manifestations are transient and manageable under close medical supervision.

### 4.7 Methodological considerations, bias, and limitations

Several limitations must be acknowledged. This analysis was designed as a pooled descriptive assessment across nine campaigns. The four study series tested heterogeneous countermeasures with different intended physiological targets (**Table 1**), and symptom data were therefore also summarized by intervention arm. Three observations limit the concern that a specific countermeasure produced the reported findings: the increase from the pre-HDT to the HDT phase was present in all eleven arms; the 28 participants who received no countermeasure at all showed a higher event rate during HDT days 1–30 than the cohort as a whole; and the differences observed between arms did not follow the intended targets of the interventions. These comparisons remain descriptive. Randomization was performed within, not across, studies, so that comparisons between studies are confounded by campaign, season and cohort; arms comprised 3 to 18 participants; and in SANS-CM the intervention arms coincide with the mode of documentation, because the lower body negative pressure and upright sitting arms were studied in the two campaigns with paper-based records and the thigh cuff and control arms in the two campaigns with digital records. The low number of baseline events in the SMC campaigns may likewise reflect differences in documentation rather than in symptom occurrence.

None of the included studies comprised a control group that did not undergo bed rest. The pre-HDT and post-HDT phases served as within-participant reference periods under otherwise identical facility conditions, including diet, climate, daily schedule and medical surveillance. Because these periods differ from the HDT phase in length and in their position within the campaign, they are not equivalent to a parallel control condition, and time-related effects such as habituation or seasonal infections cannot be excluded. Given the retrospective design, some events may therefore reflect the background incidence of head and neck symptoms in a healthy adult population rather than an effect of exposure. While the consistent increase during HDT across all campaigns and arms supports an exposure-related association, causal attribution at the level of individual symptoms remains limited.

Symptom assessment relied on retrospective CTCAE-based grading, which provides standardized classification but limits symptom-specific resolution. The intensive medical monitoring inherent to bed rest studies may have increased the detection of mild or transient symptoms and thus introduced surveillance bias, whereas symptom reporting during spaceflight is known to be subject to underreporting (Ax et al., 2025b). This limits the comparability of our data with astronaut mission logs. In the RSL-2 campaign, the medical records of four participants could not be retrieved from the archive; as the records were missing for administrative reasons unrelated to symptom status, this loss is unlikely to have introduced bias. One pre-HDT event, a cough that had begun approximately three weeks before admission and resolved two days after admission, was recorded from the participant’s history, so that its onset date precedes the observation window.

Pooling data from multiple campaigns introduces heterogeneity in protocols and in bed rest duration, although the standardized procedures of a single research facility are likely to have mitigated this effect. The healthy and comparatively young volunteer cohort limits generalizability to astronaut populations. Finally, in the absence of concurrent objective measurements, causal attribution to specific physiological mechanisms is not possible, and the mechanistic interpretations offered above should be regarded as hypothesis-generating.

### 4.8 Operational implications for spaceflight and analog research

Most head and neck symptoms observed during HDT bed rest were mild. Whether comparable low-grade manifestations accumulate into an operationally relevant symptom burden during long-duration missions cannot be inferred from analog data alone and warrants prospective assessment. New events continued to occur during the second month of bed rest, although at a lower rate than during the first, which suggests that medical monitoring should not be restricted to the initial adaptation phase. Effects on performance were not assessed, so whether head and neck symptoms affect comfort and task execution in space remains open. Preventive and supportive measures such as optimized environmental conditions, hygiene strategies and symptom-oriented self-care are low-risk options that could be evaluated prospectively. Beyond its established role in musculoskeletal and vestibular research, HDT bed rest may be a suitable setting in which to study otorhinolaryngological, ocular and dermatological responses, provided that future studies include appropriate control conditions. A further step towards strengthening the evidence base would be broader access to systematically curated in-flight medical and symptom data from space agencies. In parallel, future analog studies should integrate objective physiological readouts, including serial quantitative assessment of facial soft tissues using three-dimensional stereophotogrammetric volumetry combined with non-invasive epithelial and mucosal barrier measures across HDT and recovery, to link the symptom time course more directly with underlying mechanisms.

## Data Availability

The raw data supporting the conclusions of this article will be made available by the authors, without undue reservation.
